# A discovery about the positional distribution pattern among candidate homologous pixels and its potential application in aerial multi-view image matching

**DOI:** 10.1038/s41598-021-89501-z

**Published:** 2021-05-12

**Authors:** Ka Zhang, Wen Xiao, Yehua Sheng, Junshu Wang, Shan Zhang, Longjie Ye

**Affiliations:** 1 Key Laboratory of Virtual Geographic Environment (Nanjing Normal University), Ministry of Education, 1# WenYuan Road, Nanjing, 210023 People’s Republic of China; 2 School of Geography, Nanjing Normal University, 1# WenYuan Road, Nanjing, 210023 People’s Republic of China; 3 Jiangsu Center for Collaborative Innovation in Geographical Information Resource Development and Application, 1# WenYuan Road, Nanjing, 210023 People’s Republic of China; 4 State Key Laboratory Cultivation Base of Geographical Environment Evolution (Jiangsu Province), 1# WenYuan Road, Nanjing, 210023 People’s Republic of China; 5 Key Laboratory of Urban Land Resources Monitoring and Simulation, Ministry of Natural Resources, Shenzhen, 518034 People’s Republic of China; 6 School of Engineering, Newcastle University, Newcastle upon Tyne, NE1 7RU UK

**Keywords:** Astronomy and planetary science, Engineering, Optics and photonics

## Abstract

In aerial multi-view photogrammetry, whether there is a special positional distribution pattern among candidate homologous pixels of a matching pixel in the multi-view images? If so, can this positional pattern be used to precisely confirm the real homologous pixels? These problems have not been studied at present. Therefore, the study of the positional distribution pattern among candidate homologous pixels based on the adjustment theory in surveying is investigated in this paper. Firstly, the definition and computing method of pixel’s pseudo object-space coordinates are given, which can transform the problem of multi-view matching for confirming real homologous pixels into the problem of surveying adjustment for computing the pseudo object-space coordinates of the matching pixel. Secondly, according to the surveying adjustment theory, the standardized residual of each candidate homologous pixel of the matching pixel is figured out, and the positional distribution pattern among these candidate pixels is theoretically inferred utilizing the quantitative index of standardized residual. Lastly, actual aerial images acquired by different sensors are used to carry out experimental verification of the theoretical inference. Experimental results prove not only that there is a specific positional distribution pattern among candidate homologous pixels, but also that this positional distribution pattern can be used to develop a new object-side multi-view image matching method. The proposed study has an important reference value on resolving the defects of existing image-side multi-view matching methods at the mechanism level.

## Introduction

Extracting information of ground objects such as shape, position, size and relationship from images is a hot research topic in the fields of geosciences and computer vision^1–4^. Especially in geographic information science, aerial photogrammetry has become the main technique of 3D geospatial information collection because of its advantages of low-cost, large coverage and high efficiency^5,6^. In the process of 3D geospatial information extraction based on aerial photogrammetry, multi-view image matching is a key step. Homologous pixels of the same object in multiple images obtained from this step are fundamental for other technical steps such as automatic aerial triangulation^7,8^, production of geographic information products^9–11^ and 3D scene reconstruction^12–14^.

There are two basic problems in multi-view image matching: one is the matching measure computation, and the other is the searching scope identification for confirming the homologous pixel in each searching image^15–17^. The matching measure is the quantitative basis for judging the real homologous pixel from candidate pixels in the searching scope, which affects the accuracy and robustness of multi-view image matching. The searching scope of the homologous pixel determines how many pixels will be used as candidates in the matching process, affecting the reliability and computational efficiency. The existing multi-view image matching studies mainly focus on the following aspects: how to enhance the robustness of matching measure computation using pixels’ image-side grayscale or feature information; how to use various restriction strategies to reduce the searching scope of homologous pixel; and how to optimize the matching process.

In the aspect of matching measure computation, commonly used methods can be divided into two categories based on the type of pixels’ attributes utilized in the computing process: grayscale-based models and feature-based models. The grayscale-based matching measure models use pixels’ image-side grayscale values in red, green and blue channels to compute the similarity between two matching windows^18^. The following functions are frequently used in similarity computation, such as sum of squared differences (SSD)^19^, normalized cross correlation (NCC)^20^, zero mean normalized cross correlation (ZNCC)^21^, sum of absolute differences (SAD)^22^, and arithmetic distance^23^. The feature-based matching measure models will firstly perform a feature transform on images, such as scale invariant feature transform (SIFT)^24^, speeded up robust features transform (SURF)^25^, Harris transform^26^, Rank transform^27^, Census transform^28^, contourlet and wavelet transform^29,30^, then use obtained feature vectors as attribute values to compute the matching measure. The grayscale-based matching measure models have advantages of being simple computation and easy to realize. But they cannot work well in poorly or repetitively textured regions of the image such as water bodies and building roofs, and they are also sensitive to factors such as geometric distortions of images, changes of light intensity and contrast. In comparison, the feature-based matching measure models have strong adaptability to the changes of geometry and radiation among images. But their matching results heavily depend on the quality of feature extraction, which is more time-consuming.

In the aspect of defining the searching scope of homologous pixels, current research emphasis is on how to use various restriction strategies to reduce the searching scope and decrease the total number of candidate pixels. Frequently-used restriction strategies include pyramid hierarchy constraint^31^, epipolar line constraint^32^, prior object-space knowledge constraint, such as known elevation or DEM^33,34^, and spatial relation constraint, such as disparity continuity^35^, plane homography^36^ and geometric invariance^37,38^. Among abovementioned restriction strategies, except that the epipolar line constraint is generally workable, other constraints are only suitable to specific scenes or can be used in certain conditions. For example, the constraint of disparity continuity is not valid in disparity fault regions of ground objects (such as edges of buildings). Constraints of plane homography and spatial invariance may be invalid due to factors such as shielding relations among objects and geometric distortions of objects in the image. Furthermore, these restriction strategies can easily lead to transmission and accumulation of mismatched results, and the quality of one pixel’s matching result may be restricted by that of its neighbor pixels.

In the aspect of realization and optimization of matching process, according to the different optimizing ways, image matching methods can be divided into two types: local matching and global matching^39^. Local matching methods use the support window centered by the matching pixel to compute the matching cost, and use the strategy of “winner take all—WTA” to find the homologous pixels with the minimal cost. Local methods have advantages in computation efficiency, so can be used for real-time processing. But they mainly treat the matching problem from a local perspective, and are more sensitive to local characteristics of the image, hence can easily generate mismatches in the regions with depth discontinuity and occlusion. On the contrast, global matching methods use energy minimization to transform the image matching problem into the discrete optimization problem under the constraint of disparity continuity. Energy minimization methods such as dynamic programming algorithm^40^, belief propagation algorithm^41,42^, graph cut algorithm^43^ and Markov Random Field algorithm^44^ are often used. The overall matching results of these methods are generally good. However, these methods normally need to set many parameters with non-physical meanings, and they have high computational cost and poor real-time performance.

In addition, with the booming development of deep learning and its extensive applications in the field of geosciences^45^, deep neural networks are also applied in some stages of image matching to improve the efficiency and accuracy of existing matching methods, such as the learning of matching cost^46^, the learning of the direct process from stereopair to disparity map^47,48^. However, the methods based on neural networks still do not make any substantive improvements in the existing matching algorithms in term of the principle. Besides, they have the following obvious shortages: the matching performance depends on sufficient training data, and the trained neural network is often difficult to obtain the same matching effect when applied to unfamiliar scenes.

Overall, existing image matching methods can all be summarized as image-side methods. They only use image-side information (such as grayscale values, feature vectors, relations among pixels, etc.) of pixels in the matching window to compute the matching measure and to restrict the searching scope of homologous pixels. But the image-side information is not unique or invariable. When this information is used alone, it is difficult to construct one certain distinctive feature to accurately express the similarity between pixels, and especially difficult to precisely confirm the fine searching scope of homologous pixels. Therefore, existing matching methods cannot overcome the problem of low robustness of the matching measure caused by the phenomenon of "different spectrum of the same object" or "different objects of the same spectrum" in the image, nor the problem of the change of spatial relation among pixels caused by object occlusions and perspective distortions. When these methods are applied in difficult matching regions with repetitive or week textures, discontinuous disparity and geometric deformation, they manifest drawbacks of low efficiency and poor reliability.

Furthermore, existing research paid little attention to following questions: whether there is a certain positional distribution pattern among candidate homologous pixels of the matching pixel in multi-view images? How to quantitatively describe this positional distribution pattern? Can this pattern be used to further refine the searching scope of candidate homologous pixels, or even to exclusively confirm the homologous pixels during multi-view image matching? Thorough investigation of these questions will prospectively enhance the accuracy, efficiency and robustness of multi-view image matching from the aspect of matching mechanism, and it has the utmost significance in resolving shortcomings of existing image matching methods.

Therefore, this paper proposes a new conception of pseudo object-space coordinates and its computing model, which transforms the positional distribution pattern among candidate homologous pixels of a matching pixel into a problem of surveying adjustment. On this basis, a new method of studying the positional distribution pattern among candidate homologous pixels based on surveying adjustment theory is established, and actual aerial multi-view images are used to carry out experiments for verifying the proposed method. It is found that there is a specific corresponding relationship between the standardized residuals of candidate homologous pixels and the positional distribution pattern among these pixels, and this positional distribution pattern can be used to develop a new multi-view image matching method without utilizing the image-side grayscale information.

The remainder of the paper is organized as follows. “Method” section details the principle of the proposed method of the positional distribution pattern among candidate homologous pixels. “Experiment and discussion” section presents detailed experimental results and discussions. Finally, conclusions and potential future work are presented in “Conclusion” section.

## Method

### General idea

To study the positional distribution pattern among candidate homologous pixels of the matching pixel in multi-view images, the first thing is to transform the problem of seeking homologous pixels into a problem of surveying adjustment. As all know, in photogrammetry, although the image-space results of multi-view image matching are a group of homologous pixels identified from many candidate pixels in multiple target images corresponding to the matching pixel in the reference image, the final aim is to compute the object-space 3D geospatial coordinates of the ground object using image-space 2D row and column coordinates of the homologous pixels. Therefore, considering the fact that the ground object corresponding to the homologous pixels has unique object-space 3D coordinates, this paper proposes a new concept of pseudo object-space coordinates to express the object-space information computed with all candidate pixels referring to the conception of pseudo distance in GNSS data processing. Based on this new concept, the paper transforms the problem of multi-view image matching into a problem of surveying adjustment of pseudo object-space coordinates according to the theories of surveying error adjustment.

**Figure 1:**
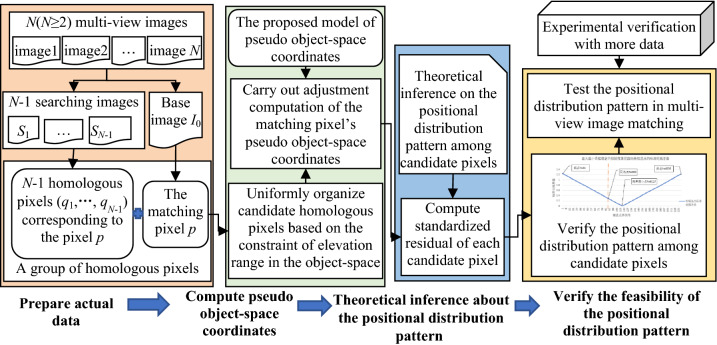
The general flow diagram of the proposed research.

The diagram of the proposed method is shown in Fig. 1. Firstly, experimental data are prepared as follows: *N* images with known interior and exterior orientation parameters (*N* ≥ 2 is the total number of multi-view images), and one group of real homologous pixels in these images (one pixel is used as the matching pixel). Secondly, based on the proposed model of pseudo object-space coordinates, adjustment of the matching pixel’s pseudo object-space coordinates is carried out. Thirdly, according to the adjustment results of pseudo object-space coordinates, the standardized residual of each candidate pixel in the searching scope of homologous pixels is computed. Fourthly, based on the fact that errors in observations will strongly distort the adjustment results, the standardized residual is used as a quantitative index to depict the positional feature of each candidate pixel, and a theoretical inference about the positional distribution pattern among candidate homologous pixels based on the surveying adjustment theory is hypothesized. Lastly, the experimental data are used to draw the curve of the relationship between the position of each candidate pixel and its standardized residual for verifying the inference, and to further test the application effect of the positional distribution pattern in multi-view image matching.

To describe the subsequent content conveniently, for *N* (*N* ≥ 2) multiple images with known interior and exterior orientation parameters obtained by automatic aerotriangulation software, one of *N* images used to pick the matching pixel is named as the base image *I*_0_, and the remaining *N*−1 images as searching images *I*_1_, …, *I*_*k*_, …, *I*_*N*-1_ (*k* = 1, 2, …, *N*−1). Projection centers of the base image and searching images are denoted as *S*_0_ and *S*_*k*_, and respective exterior orientation parameters are denoted as (*X*_0_, *Y*_0_, *Z*_0_, *φ*_0_, *ω*_0_, *κ*_0_) and (*X*_*k*_, *Y*_*k*_, *Z*_*k*_, *φ*_*k*_, *ω*_*k*_, *κ*_*k*_). The row and column numbers of the pixel are denoted as (*r*, *c*), and corresponding image plane coordinates are (*x*, *y*).

### Definition and computation of pseudo object-space coordinates

The definition of proposed pseudo object-space coordinates is as follows: for a matching pixel *p* in the base image *I*_0_, the 3D coordinates, which are calculated with the pixel *p* and all candidate pixels in the searching scope of its homologous pixels in the *N*−1 searching images, are defined as the pseudo object-space coordinates of the pixel *p*. Seen from the definition, the pseudo object-space coordinates have the form of 3D coordinates (*X*, *Y*, *Z*), but are not true 3D coordinates of ground object corresponding to the pixel *p*, hence named as pseudo coordinates.

**Figure 2:**
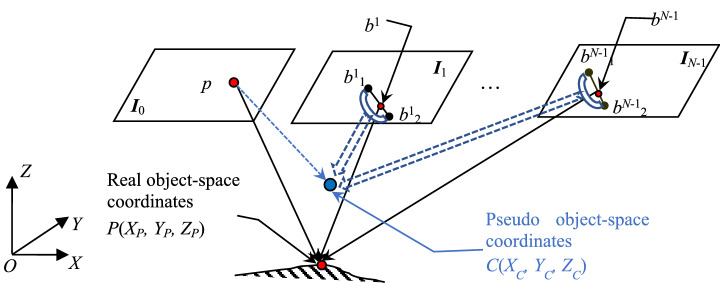
The relation between pixel’s real object-space 3D coordinates and its pseudo object-space coordinates.

The sketch for displaying the relation between a pixel’s real object-space 3D coordinates and its pseudo object-space coordinates is shown in Fig. 2. In this figure, the point *p* represents the matching pixel in the base image, and its real homologous pixels in *N*−1 searching images are denoted as [*b*^1^, …, *b*^*N*−1^]. Two endpoints of the searching range of homologous pixel in the *k*th searching image are marked as *b*^*k*^_1_ and *b*^*k*^_2_ (that are on the epipolar line, and the method of confirming which can be referred to in “Analysis of the positional distribution pattern among candidate homologous pixels based on the adjustment of pseudo object-space coordinates”), and the *j*th candidate pixel on this epipolar line is labeled as *b*^*k*^_*j*_ . Then, the coordinates (*X*_*P*_*, Y*_*P*_*, Z*_*P*_) computed by the pixel *p* and its real homologous pixels [*b*^1^, …, *b*^*N*-1^] are real object-space 3D coordinates, and the coordinates (*X*_*C*_*, Y*_*C*_*, Z*_*C*_) computed by the pixel *p* and all its candidate pixels {[*b*^1^_1_, …, *b*^1^_*j*_, …, *b*^1^_2_], …, [*b*^*N*−1^_1_, …, *b*^*N*−1^_*j*_, …, *b*^*N*−1^_2_]} in *N*−1 searching images are pseudo object-space coordinates. In addition, the dashed arrow pointing to the pseudo coordinates is used to represent the participated matching pixel and its candidate homologous pixels, and the hollow arc on each searching image is used to represent all candidate pixels.

To compute the pseudo object-space coordinates, the method of multi-view forward intersection in photogrammetry is used. For the matching pixel *p* and its ${M}_{t}=\sum_{k=1}^{N-1}{M}_{k}$ candidate homologous pixels in *N*−1 searching images (*M*_*k*_ is the total number of candidate homologous pixels in the *k*th searching image *I*_*k*_), the pseudo object-space coordinates of pixel *p* is computed by the following steps: firstly, the error equation matrix used to solve the pseudo object-space coordinates is formed as **V** = **AX** − **L** according to the principle of multi-view forward intersection. In this error equation matrix, **V** is the residual matrix of 2(*M*_*t*_ + 1) image plane coordinates (its dimensions are 2(*M*_*t*_ + 1) rows × 1 column); **X** = [*dX*, *dY*, *dZ*]^*T*^ is the coordinate corrections matrix of undetermined pseudo object-space coordinates (its dimensions are 3 rows × 1 column); **A** and **L** are the coefficient matrix and the constant matrix (their dimensions are 2(*M*_*t*_ + 1) rows × 3 columns and 2(*M*_*t*_ + 1) rows × 1 column respectively), and detailed formulas for computing the elements in each row of **A** and **L** can be referred to in any photogrammetry textbooks^49^. Secondly, using the principle of least squares, the error equation matrix is solved by equation **X** = (**A**^*T*^**A**)^−1^(**A**^*T*^**L**) to obtain the corrections (*dX*, *dY*, *dZ*) of the initial coordinates (*X*^0^, *Y*^0^, *Z*^0^). Then, the pseudo object-space coordinates (*X*, *Y*, *Z*), of the pixel *p*, are obtained by add the corrections: *X* = *X*^0^ + *dX*, *Y* = *Y*^0^ + *dY* and *Z* = *Z*^0^ + *dZ*.

To be noted, although the computing of pseudo object-space coordinates is based on the principle of multi-view forward intersection, their computing processes are not identical. In traditional multi-view forward intersection, only one candidate pixel in each searching image is used in the computing process, and this process needs to run iteratively. However, in the computing of pseudo object-space coordinates, all candidate homologous pixels in the searching range of each searching image corresponding to the matching pixel are used, and corrections of undetermined coordinates only need to be calculated once. The goal of computing pseudo object-space coordinates is not to solve the real object-space 3D coordinates of the matching pixel, but to analyze the positional distribution pattern among candidate homologous pixels according to the adjustment results of pseudo object-space coordinates.

### Analysis of the positional distribution pattern among candidate homologous pixels based on the adjustment of pseudo object-space coordinates

Seen from above, for a matching pixel in the base image, there is a certain correlation between the computation of its pseudo object-space coordinates and the multi-view image matching of confirming its homologous pixels. That is, they both need the participation of all candidate homologous pixels in the searching range of each searching image. So, the problem of multi-view image matching can be transformed into a surveying adjustment problem of pseudo object-space coordinates to analyze the positional distribution pattern among candidate homologous pixels in the multi-view images.

#### Theoretical inference on the positional distribution pattern among candidate homologous pixels

**Figure 3:**
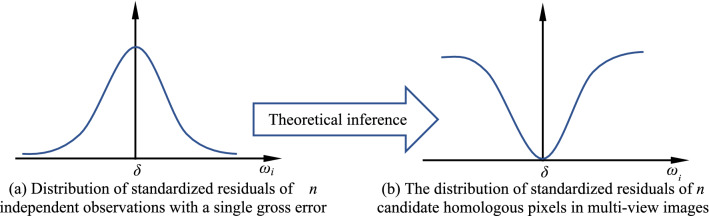
The theoretical inference on the positional distribution pattern among candidate homologous pixels in multi-view images according to the surveying adjustment theory.

According to the reliability theory of surveying adjustment system^50^, for *n* independent observations with a single gross error, the standardized residual *ω*_*i*_ (*i* = 1,2,…,*n*) of each observation after adjustment obeys the normal distribution with mean value *δ* and variance 1, and its distribution is in the shape of a Gaussian bell (shown in Fig. 3a). If this theory is applied to the adjustment process of the matching pixel’s pseudo object-space coordinates, and the group of real homologous pixels in all candidate pixels corresponding to the matching pixel is seen as one “special” gross error, then the problem of multi-view image matching can be regarded as an “special” surveying adjustment problem using *n* independent observations with a single gross error. Therefore, the following theoretical inference about the positional distribution pattern among candidate homologous pixels in multi-view images is made: after adjustment of pseudo object-space coordinates, the standardized residuals of all candidate homologous pixels will also obey one certain distribution pattern, which is in the form of an opposite shape of Fig. 3a, like an approximate V-shaped distribution (shown in Fig. 3b).

#### Computation of standardized residuals for candidate homologous pixels

Based on the above theoretical inference, to analyze the positional distribution pattern among candidate homologous pixels of a matching pixel, their standardized residuals should be firstly calculated. The computing process is described as follows: (1) For matching pixel *p*, the searching range of its candidate homologous pixels in each searching image is identified under the constraint of a predefined elevation range in the object-space; (2) For different searching ranges in different searching images, their lengths are refined to a uniform value for uniformly assigning independent candidate homologous pixels in different searching images to different groups. (3) The adjustment computation of pseudo object-space coordinates of the matching pixel is carried out to obtain the standardized residual of each group of candidate homologous pixels.

##### Determining candidate homologous pixels for the matching pixel based on the constraint of elevation range in object space

**Figure 4:**
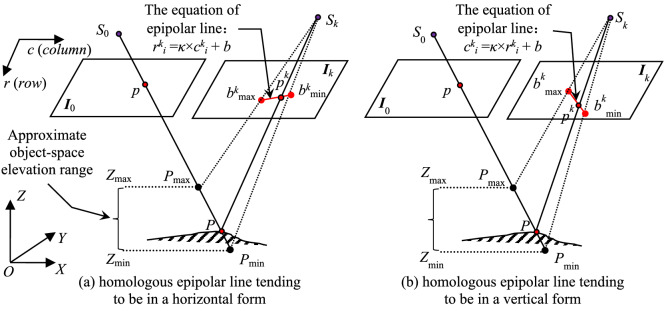
The sketch of determining candidate homologous pixels based on the object-space elevation range.

As shown in Fig. 4, for matching pixel *p* in base image *I*_0_, its candidate homologous pixels in each searching image are determined by the following method under the constraint of object-space approximate elevation range.

① According to a priori knowledge interactively input by the operator, the approximate object-space minimum and maximum elevations *Z*_min_ and *Z*_max_ are assigned to the base image. It is not necessary to be accurate, just to ensure that the range [*Z*_min_, *Z*_max_] includes the real elevation of the matching pixel *p*. Then the ground point *P* corresponding to the pixel *p* must locate between the line segment *P*_min_*P*_max_ along the projection line *S*_0_*P*. Utilizing the collinearity equation (shown in Eq. (1)), object-space plane coordinates (*X*_min_, *Y*_min_) and (*X*_max_, *Y*_max_) of the points *P*_min_ and *P*_max_ can be figured out by the image plane coordinates of pixel *p* and elevations *Z*_min_ and *Z*_max_,

1 $$\left\{\begin{array}{c}X={X}_{0}+(Z-{Z}_{0})\frac{{a}_{1}^{0}x+{a}_{2}^{0}y-{a}_{3}^{0}f}{{c}_{1}^{0}x+{c}_{2}^{0}y-{c}_{3}^{0}f}\\ Y={Y}_{0}+(Z-{Z}_{0})\frac{{b}_{1}^{0}x+{b}_{2}^{0}y-{b}_{3}^{0}f}{{c}_{1}^{0}x+{c}_{2}^{0}y-{c}_{3}^{0}f}\end{array}\right.$$

where, *f* is the focal length in the camera’s interior orientation parameters; coefficients (*a*^0^_1_, *a*^0^_2_, *a*^0^_3_; *b*^0^_1_, *b*^0^_2_, *b*^0^_3_; *c*^0^_1_, *c*^0^_2_, *c*^0^_3_) are nine direction cosines in the rotation matrix determined by the exterior rotational angles (*φ*_0_, *ω*_0_, *κ*_0_) of the base image.

② Utilizing the collinearity equation (shown in Eq. (2)), the object-space points *P*_min_(*X*_min_, *Y*_min_, *Z*_min_) and *P*_max_(*X*_max_, *Y*_max_, *Z*_max_) are re-projected onto the *k*th searching image *I*_*k*_ to obtain the corresponding pixels *b*^*k*^_min_(*x*^*k*^_min_, *y*^*k*^_min_) and *b*^*k*^_max_(*x*^*k*^_max_, *y*^*k*^_max_). The homologous pixel *p*^*k*^ of the pixel *p* in the image *I*_*k*_ must locate on the line segment *b*^*k*^_min_*b*^*k*^_max_, which is actually a segment of the homologous epipolar line. The segment *b*^*k*^_min_*b*^*k*^_max_ is the searching range of the homologous pixel *p*^*k*^ in the image *I*_*k*_, and all *M*_*k*_ pixels located on *b*^*k*^_min_*b*^*k*^_max_ are considered as candidate homologous pixels. Next, according to the camera’s interior orientation parameters, row and column numbers (*r*^*k*^_min_, *c*^*k*^_min_) and (*r*^*k*^_max_, *c*^*k*^_max_) of the points *b*^*k*^_min_ and *b*^*k*^_max_ are figured out. Then the linear equation of epipolar line *b*^*k*^_min_*b*^*k*^_max_ can be defined, based on which the row and column numbers (*r*^*k*^_*i*_, *c*^*k*^_*i*_) of the *i*th candidate pixel *p*^*k*^_*i*_ in the image *I*_*k*_ are obtained (*i* = 1, 2, …, *M*_*k*_).

2 $$\left\{\begin{array}{c}x=-f\frac{{a}_{1}\left(X-{X}_{k}\right)+{b}_{1}\left(Y-{Y}_{k}\right)+{c}_{1}(Z-{Z}_{k})}{{a}_{3}\left(X-{X}_{k}\right)+{b}_{3}\left(Y-{Y}_{k}\right)+{c}_{3}(Z-{Z}_{k})}\\ y=-f\frac{{a}_{2}\left(X-{X}_{k}\right)+{b}_{2}\left(Y-{Y}_{k}\right)+{c}_{2}(Z-{Z}_{k})}{{a}_{3}\left(X-{X}_{k}\right)+{b}_{3}\left(Y-{Y}_{k}\right)+{c}_{3}(Z-{Z}_{k})}\end{array}\right.$$

where, coefficients (*a*_1_, *a*_2_, *a*_3_; *b*_1_, *b*_2_, *b*_3_; *c*_1_, *c*_2_, *c*_3_) are nine direction cosines in the rotation matrix determined by the exterior rotational angles (*φ*_*k*_, *ω*_*k*_, *κ*_*k*_) of the *k*th searching image *I*_*k*_; (*X*_*k*_, *Y*_*k*_, *Z*_*k*_) are object-space 3D coordinates of the projection center of the image *I*_*k*_.

In Fig. 4, there are two cases of the form of epipolar line in each searching image, and the linear equation of each case is confirmed as follows. In following description, *int*() is the integral function, *abs*() is the absolute value function, *min*() is the minimum function, and *max*() is the maximum function.

- If *abs*(*c*^*k*^_max_ − *c*^*k*^_min_) ≥ *abs*(*r*^*k*^_max_ − *r*^*k*^_min_), the epipolar line tends to be horizontal (shown in Fig. 4a), and its linear equation is *r*^*k*^_*i*_ = *κ* × *c*^*k*^_*i*_ + *b*. Where, *κ* and *b* are respectively the slope and intercept of the line, and are computed by equations *κ* = (*r*^*k*^_max_ − *r*^*k*^_min_)/(*c*^*k*^_max_ − *c*^*k*^_min_) and *b* = *r*^*k*^_max_ − *κ* × *c*^*k*^_max_. The range of values of variable *c*^*k*^_*i*_ is [*min*(*c*^*k*^_max_, *c*^*k*^_min_), *max*(*c*^*k*^_max_, *c*^*k*^_min_)], and the total number of candidate homologous pixels is computed by equation *M*_*k*_ = *int*(*abs*(*c*^*k*^_max_ − *c*^*k*^_min_) + 1).
- If *abs*(*c*^*k*^_max_ − *c*^*k*^_min_) < *abs*(*r*^*k*^_max_ − *r*^*k*^_min_), the epipolar line tends to be vertical (shown in Fig. 4b), and its linear equation is *c*^*k*^_*i*_ = *κ* × *r*^*k*^_*i*_ + *b*. Corresponding slope and intercept of the line are computed by equations *κ* = (*c*^*k*^_max_ − *c*^*k*^_min_)/(*r*^*k*^_max_ − *r*^*k*^_min_) and *b* = *c*^*k*^_max_ − *κ* × *r*^*k*^_max_. The range of values of variable *r*^*k*^_*i*_ is [*min*(*r*^*k*^_max_, *r*^*k*^_min_), *max*(*r*^*k*^_max_, *r*^*k*^_min_)], and the total number is computed by equation *M*_*k*_ = *int*(*abs*(*r*^*k*^_max_ − *r*^*k*^_min_) + 1).

##### Uniformly grouping candidate homologous pixels in searching images

**Figure 5:**
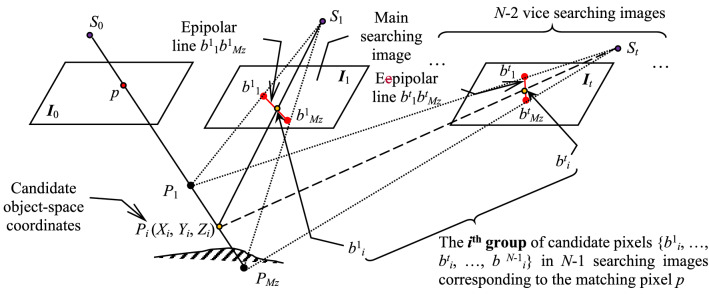
The sketch of uniformly grouping candidate homologous pixels in multi-view images.

As seen from above description, for the matching pixel *p*, the total number *M*_*k*_ of its candidate homologous pixels in each searching image are different. Furthermore, different candidate pixels in different searching images are independent from each other, and it is difficult to construct any relations among these pixels utilizing their serial numbers. Thus, to analyze the distribution pattern among candidate homologous pixels, the total number of candidate homologous pixels in each searching image is unified by the following method, which can uniformly assign independent candidate pixels to different groups (the sketch is shown in Fig. 5).

① According to the number of candidate homologous pixels in each searching image, the image with the maximal number is used as the main searching image (like image *I*_1_ in Fig. 5), and remaining *N*−2 images are named as vice searching image *I*_*t*_ (*t* = 2, …, *N*−1). The total number of candidate homologous pixels in the main searching image is labeled as *M*_*z*_, and its value is computed by the equation *M*_*z*_ = *max*(*M*_*k*_ |*k* = 1, 2,…, *N*−1).

② For the *i*th candidate pixel *b*^1^_*i*_(*r*^1^_*i*_, *c*^1^_*i*_) on the homologous epipolar line *b*^1^_1_*b*^1^_*Mz*_ in the main searching image (*i* = 1, 2,…, *M*_*z*_), the candidate object-space coordinates (*X*_*i*_, *Y*_*i*_, *Z*_*i*_) are firstly figured out with the pixels *p* and *b*^1^_*i*_. Next, using the collinearity equation and the linear equation of homologous epipolar line *b*^*t*^_1_*b*^*t*^_*Mz*_ in the *t*th vice searching image *I*_*t*_, the point *P*_*i*_ is re-projected onto the image *I*_*t*_ to yield the candidate pixel *b*^*t*^_*i*_ corresponding to the pixel *b*^1^_*i*_. Then these pixels {*b*^1^_*i*_, …, *b*^*t*^_*i*_, …, *b*^*N*-1^_*i*_} are organized as the *i*th group of candidate homologous pixels of the pixel *p*.

③ After all candidate pixels in the main searching image are processed by the step 2, the number of candidate homologous pixels corresponding to the matching pixel *p* in each searching image will have the same value *M*_*z*_, and the candidate pixels with the same serial number belong to the same group. Therefore, the original independent candidate pixels in all searching images is unified into the following *M*_*z*_ groups: {*b*^1^_1_, *b*^2^_1_, …, *b*^*N*-1^_1_}, {*b*^1^_2_, *b*^2^_2_, …, *b*^*N*-1^_2_}, …, {*b*^1^_*i*_, *b*^2^_*i*_, …, *b*^*N*-1^_*i*_}, …, {*b*^1^_*Mz*_, *b*^2^_*Mz*_, …, *b*^*N*-1^_*Mz*_} (the group serial number *i* is from 1 to *M*_*z*_).

 To be noted, after completing the unifying process of candidate pixels, except for the main searching image, there are repetitive pixels in *M*_*z*_ candidate pixels {*b*^*t*^_1_, *b*^*t*^_2_, …, *b*^*t*^_*Mz*_} in the *t*th vice searching image *I*_*t*_, and repetitive pixels must be neighbor pixels with consecutive serial numbers.

##### Computing standardized residual for each group of candidate homologous pixels based on surveying adjustment theory

According to the surveying adjustment theory, utilizing relevant information during the adjustment of pseudo object-space coordinates, the standardized residual of each candidate homologous pixel can be figured out and used to analyze the positional distribution pattern among candidate pixels. For *M*_*z*_ groups of candidate homologous pixels in searching images: {*b*^1^_1_, *b*^2^_1_, …, *b*^*N*-1^_1_}, …, {*b*^1^_*Mz*_, *b*^2^_*Mz*_, …, *b*^*N*-1^_*Mz*_} corresponding to the matching pixel *p*, the standardized residual *ω*_*i*_ of the *i*th group of candidate pixels {*b*^1^_*i*_, *b*^2^_*i*_, …, *b*^*N*-1^_*i*_} is computed as follows:

① Removing repetitive candidate homologous pixels in the searching image. For the *M*_*z*_ candidate pixels {*b*^*k*^_1_, …, *b*^*k*^_*j*_, …, *b*^*k*^_*Mz*_} (*j* = 1, …, *M*_*z*_) in the *k*th searching image *I*_*k*_ (*k* = 1, …, *N*−1), starting from the second candidate pixel, each pixel is judged whether it repeats with the previous adjacent pixel one by one. If yes, it will be marked as a repetitive pixel. After all *M*_*z*_ candidate pixels are processed by the repeatability judgement,${\overline{M} }_{k}$(${\overline{M} }_{k}$≤*M*_*z*_) new candidate pixels {*q*^*k*^_1_, …, *q*^*k*^_*j’*_, …,${q}_{{\overline{M} }_{k}}^{k}$} (*jˈ* = 1, …,${\overline{M} }_{k}$) without repetitive pixels are obtained, and the sum of the matching pixel *p* and its candidate homologous pixels is *M*:$M=(1+{\sum }_{k=1}^{N-1}{\overline{M} }_{k})$. Furthermore, the correspondence relation between the original serial number *j* and the new serial number *jˈ* is known.

② Constructing the error equation matrix of all *M* pixels. According to the computing of pseudo object-space coordinates in "Definition and computation of pseudo object-space coordinates", for the matching pixel and its ${\overline{M} }_{k}$ candidate homologous pixels in each searching image, the error equation matrix (shown in Eq. (3)) for solving the pseudo object-space coordinates of the pixel *p* can be formed.

3 $${\mathbf{V}}_{2M\times 1}={\mathbf{A}}_{2M\times 3}{\mathbf{X}}_{3\times 1}-{\mathbf{L}}_{2M\times 1}$$

Detailed forms of matrixes **A**_2*M*×3_ and **L**_2*M*×1_ are shown in Eq. (4). Where, the superscript of each element (*I*_0_, *I*_1_, …, *I*_*N*-1_) represents the label of the image, and the number from 1 to ${\overline{M} }_{1}$ or ${\overline{M} }_{N-1}$ in the subscript of each element represents the serial number of candidate homologous pixel in different searching image.

4 $$\left\{\begin{array}{ll}{\mathbf{V}}_{2M\times 1}={\left[{v}_{x}^{{I}_{0}},{v}_{y}^{{I}_{0}},{v}_{x,1}^{{I}_{1}},{v}_{y,1}^{{I}_{1}},\cdots ,{v}_{x,{M^{\prime}}_{1}}^{{I}_{1}},{v}_{y,{M^{\prime}}_{1}}^{{I}_{1}},\cdots ,{v}_{x,1}^{{I}_{N-1}},{v}_{y,1}^{{I}_{N-1}},\cdots ,{v}_{x,{\overline{M} }_{N-1}}^{{I}_{N-1}},{v}_{y,{\overline{M} }_{N-1}}^{{I}_{N-1}}\right]}^{T}\\ {\mathbf{X}}_{3\times 1}={\left[\begin{array}{ccc}dX& dY& dZ\end{array}\right]}^{{\varvec{T}}}\\ {\mathbf{A}}_{2M\times 3}=\left[\begin{array}{c}\begin{array}{ccc}{a}_{11}^{{I}_{0}}& {a}_{12}^{{I}_{0}}& {a}_{13}^{{I}_{0}}\end{array}\\ \begin{array}{ccc}{a}_{21}^{{I}_{0}}& {a}_{22}^{{I}_{0}}& {a}_{23}^{{I}_{0}}\end{array}\\ \begin{array}{ccc}{a}_{{11,1}}^{{I}_{1}}& {a}_{{12,1}}^{{I}_{1}}& {a}_{{13,1}}^{{I}_{1}}\end{array}\\ \begin{array}{ccc}{a}_{{21,1}}^{{I}_{1}}& {a}_{{22,1}}^{{I}_{1}}& {a}_{{23,1}}^{{I}_{1}}\end{array}\\ \vdots \\ \begin{array}{ccc}{a}_{11,{\overline{M} }_{1}}^{{I}_{1}}& {a}_{12,{\overline{M} }_{1}}^{{I}_{1}}& {a}_{13,{\overline{M} }_{1}}^{{I}_{1}}\end{array}\\ \begin{array}{ccc}{a}_{21,{\overline{M} }_{1}}^{{I}_{1}}& {a}_{22,{\overline{M} }_{1}}^{{I}_{1}}& {a}_{23,{\overline{M} }_{1}}^{{I}_{1}}\end{array}\\ \vdots \\ \begin{array}{ccc}{a}_{{11,1}}^{{I}_{N-1}}& {a}_{{12,1}}^{{I}_{N-1}}& {a}_{{13,1}}^{{I}_{N-1}}\end{array}\\ \begin{array}{ccc}{a}_{{21,1}}^{{I}_{{N}_{k}}}& {a}_{{22,1}}^{{I}_{{N}_{k}}}& {a}_{{23,1}}^{{I}_{{N}_{k}}}\end{array}\\ \vdots \\ \begin{array}{ccc}{a}_{11,{\overline{M} }_{N-1}}^{{I}_{N-1}}& {a}_{12,{\overline{M} }_{N-1}}^{{I}_{N-1}}& {a}_{13,{\overline{M} }_{N-1}}^{{I}_{N-1}}\end{array}\\ \begin{array}{ccc}{a}_{21,{\overline{M} }_{N-1}}^{{I}_{N-1}}& {a}_{22,{\overline{M} }_{N-1}}^{{I}_{N-1}}& {a}_{23,{\overline{M} }_{N-1}}^{{I}_{N-1}}\end{array}\end{array}\right],{\mathbf{L}}_{2M\times 1}=\left[\begin{array}{c}{x}^{{I}_{0}}-{\left({x}^{{I}_{0}}\right)}^{0}\\ {y}^{{I}_{0}}-{\left({y}^{{I}_{0}}\right)}^{0}\\ {x}_{1}^{{I}_{1}}-{\left({x}_{1}^{{I}_{1}}\right)}^{0}\\ {y}_{1}^{{I}_{1}}-{\left({y}_{1}^{{I}_{1}}\right)}^{0}\\ \vdots \\ {x}_{{M^{\prime}}_{1}}^{{I}_{1}}-{\left({x}_{{\overline{M} }_{1}}^{{I}_{1}}\right)}^{0}\\ {y}_{{M^{\prime}}_{1}}^{{I}_{1}}-{\left({y}_{{\overline{M} }_{1}}^{{I}_{1}}\right)}^{0}\\ \vdots \\ {x}_{1}^{{I}_{N-1}}-{\left({x}_{1}^{{I}_{N-1}}\right)}^{0}\\ {y}_{1}^{{I}_{N-1}}-{\left({y}_{1}^{{I}_{N-1}}\right)}^{0}\\ \vdots \\ {x}_{{\overline{M} }_{N-1}}^{{I}_{N-1}}-{\left({x}_{{\overline{M} }_{N-1}}^{{I}_{N-1}}\right)}^{0}\\ {y}_{{\overline{M} }_{N-1}}^{{I}_{N-1}}-{\left({y}_{{\overline{M} }_{N-1}}^{{I}_{N-1}}\right)}^{0}\end{array}\right]\end{array}\right.$$

③ Computing the reliability matrix. Utilizing the coefficient matrix **A**, the constant matrix **L**, and the weight matrix **P**_*ll*_ of observations (as all observations in this paper are image plane coordinates of pixels and are obtained independently, the weight matrix is a unit matrix **E**), the reliability matrix **R** can be derived by the following equation.

5 $${\mathbf{R}}_{2M\times 2M}=\left({\mathbf{P}}_{{\varvec{l}}l}^{-1}-\mathbf{A}{\left({\mathbf{A}}^{T}{\mathbf{P}}_{ll}\mathbf{A}\right)}^{-1}{\mathbf{A}}^{T}\right){\mathbf{P}}_{ll}$$

④ Resolving the error equation matrix. Based on the principle of least squares, the error equation matrix shown in Eq. (3) is resolved to obtain the coordinates correction matrix **X**, the residual matrix **V** of all observations and the root mean square error of unit weight *σ*_0_.

6 $$\left\{\begin{array}{c}X={\left({\mathbf{A}}^{T}{\mathbf{P}}_{ll}\mathbf{A}\right)}^{-1}\left({\mathbf{A}}^{T}{\mathbf{P}}_{ll}\mathbf{L}\right)\\ V=AX-L\\ {\sigma }_{0}=\sqrt{{\mathbf{V}}^{T}{\mathbf{P}}_{ll}\mathbf{V}/\left(2M-3\right)}\end{array}\right.$$

⑤ Computing the standardized residual *ω*_*n*_ of the *n*th candidate pixel in all *M* pixels (*n* ∈ [1, *M*]). As there are two observations of one pixel at the *x* and *y* coordinate directions, the standardized residual of each candidate pixel is computed by taking the average of standardized residuals of observations *x* and *y*, as shown in Eq. (7).

7 $${\omega }_{n}=\left(\frac{abs\left({\left({\mathbf{V}}_{ll}\right)}_{2{\varvec{n}}, 0}\right)}{\sqrt{{\left(\mathbf{R}\right)}_{2{\varvec{n}},2{\varvec{n}}}}\times {\sigma }_{0}/\sqrt{{\left({\mathbf{P}}_{ll}\right)}_{2{\varvec{n}},2n}}}+\frac{abs\left({\left({\mathbf{V}}_{ll}\right)}_{2n+{1,0}}\right)}{\sqrt{{\left(\mathbf{R}\right)}_{2n+{1,2}n+1}}\times {\sigma }_{0}/\sqrt{{\left({\mathbf{P}}_{ll}\right)}_{2n+{1,2}n+1}}}\right)/2$$

⑥ Computing the standardized residual *ω*(*b*^*k*^_*j*_) of the *j*th candidate pixel *b*^*k*^_*j*_ in the *k*th searching image *I*_*k*_. Known from the repeatability judgement of the step (1), serial number *j* of pixel *b*^*k*^_*j*_ corresponds to serial number *jˈ* in the new candidate pixels without repetitive pixels. In addition, the total number of the matching pixel and its candidate pixels in (*k*−1) searching images processed by the repetitiveness assessment is *Mˈ*:$M{^{\prime}}=(1+{\sum_{{i}^{^{\prime}}=1}^{k-1}\overline{M} }_{{i}^{^{\prime}}})$. So, *ω*(*b*^*k*^_*j*_) is computed as follows.

8 $$\omega \left({b}_{j}^{k}\right)={\omega }_{{M}^{^{\prime}}+{j}^{^{\prime}}}$$

⑦ Computing the standardized residual *ω*_*i*_ of the *i*th group of candidate homologous pixels. As the *i*th group of candidate pixels consist of *N*−1 candidate pixels, *ω*_*i*_ is computed by taking the average of standardized residuals of *N*−1 candidate pixels, as shown in Eq. (9).

9 $${\omega }_{i}=\frac{\sum_{k=1}^{N-1}\omega \left({b}_{i}^{k}\right)}{N-1}$$

## Experiment and discussion

### Experimental data

**Figure 6:**
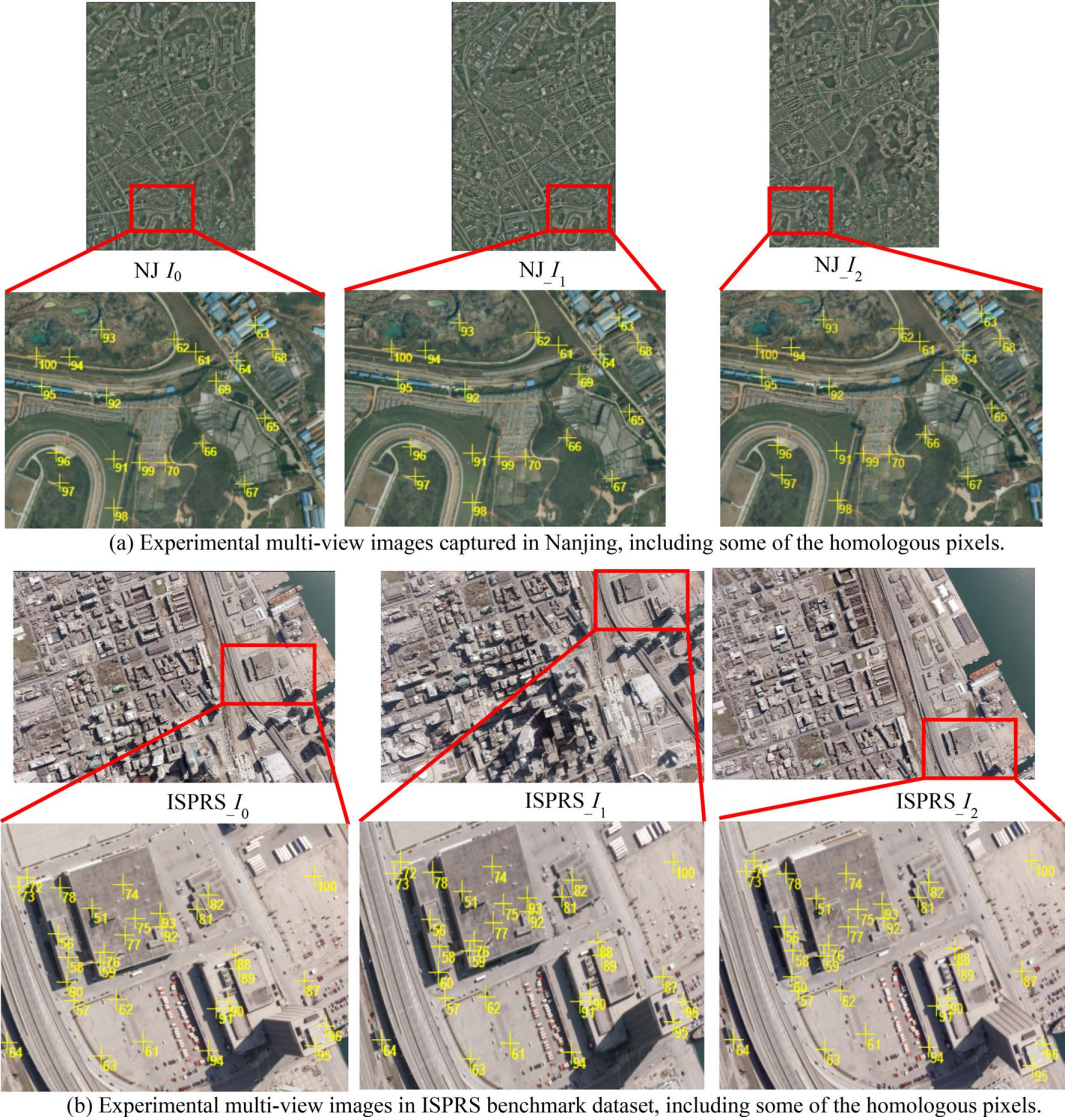
Two types of experimental aerial images (Images in sub-figure (**a**) were captured by our partner Jiangsu Digitalland Technology Corporation, Ltd., Co. with an UltraCam metric digital camera in Nanjing China in November 2011, and images in sub-figure (**b**) are internationally public benchmark data in Toronto Canada provided by ISPRS Working Group III/4: http://www2.isprs.org/commissions/comm3/wg4).

To verify the proposed approach, experiments were carried out using two sets of actual aerial multi-view images. Figure 6a shows three aerial images of Nanjing, China (captured by Jiangsu Digitalland Technology Corporation, Ltd., Co., using an UltraCam metric digital camera in November 2011), and Fig. 6b shows three aerial images of Toronto, Canada (public data in the ISPRS benchmark dataset, obtained through the ISPRS official website: http://www2.isprs.org/commissions/comm3/wg4). For the two datasets, the base image and two searching images are respectively labeled as NJ___*I*_0_, NJ___*I*_1_, NJ___*I*_2_ and ISPRS___*I*_0_, ISPRS___*I*_1_, ISPRS___*I*_2_, the approximate elevation ranges of the region covered by these images are respectively [− 28 m, 188 m] and [− 20 m, 210 m], the forward overlap of these images is around 60%, and their interior and exterior orientation parameters are all known (detailed parameters are listed in Tables 1 and 2). In addition, 100 groups of actual homologous pixels were semi-automatically selected by an image-side multi-view matching method based on NCC^10^ for each experimental data, and were used as ground truth for the following two experiments (Part of matching pixels in the base image and their homologous pixels in the two searching images are depicted in Fig. 6. The yellow cross represents their positions, and the number beside the cross is the serial number). Image-space row and column numbers and object-space 3D coordinates of these homologous pixels are respectively labeled as (*r*^*k*^_*j*_, *c*^*k*^_*j*_) and (*X*_*j*_, *Y*_*j*_, *Z*_*j*_) (*k* = 0, *k* = 1, *k* = 2 is respectively the serial number of the based image, the first and the second searching images; and *j* = 1, 2, …, 100).

**Table 1:** Interior orientation parameters of experimental aerial images.

Image type	Image-space resolution [pixel]	Focal length [mm]	The position of principal point [pixel]		Pixel size [mm]
			Row	Column	
Nanjing aerial images	11,310 columns × 17,310 rows	70.5	8634	5654	0.006
ISPRS benchmark images	11,500 columns × 7500 rows	101.4	3769	5749	0.009

**Table 2:** Exterior orientation parameters of experimental aerial images.

Image type and tagID		WGS-84 coordinates of projection centres			exterior rotational angles		
		*X*[m]	*Y*[m]	*Z*[m]	*φ* [rad]	*ω*[rad]	*κ*[rad]
Nanjing aerial images	NJ___*I*_0_	397,361.04579	3,552,859.53939	3718.27647	− 0.008873	0.015662	− 0.014234
	NJ___*I*_1_	396,163.27625	3,552,863.48668	3718.31694	− 0.007112	0.009475	− 0.011622
	NJ___*I*_2_	398,558.69302	3,552,849.68689	3718.66453	− 0.008784	0.015934	− 0.017909
ISPRS benchmark images	ISPRS___*I*_0_	631,179.232	4,834,062.777	1636.415	0.001505	0.000377	− 1.751372
	ISPRS___*I*_1_	630,789.997	4,834,064.034	1632.821	0.002248	0.000534	− 1.748176
	ISPRS___*I*_2_	631,567.432	4,834,062.247	1640.148	0.000473	0.000852	− 1.749056

### Experimental design

Utilizing above data, two experiments were designed as follows: experiment **I** was used to verify the theoretical inference on the positional distribution pattern among candidate homologous pixels, and experiment **II** was used to further test whether the positional distribution pattern can be applied in multi-view image matching.

Experiment **I**: For each matching pixel in the base image, firstly its initial set of *m* groups of candidate homologous pixels in the two searching images are confirmed and their standardized residuals computed, and the standardized residual curve is depicted to illustrate the positional distribution form among candidate homologous pixels. Secondly, if the curve of the initial set of candidate homologous pixels obeys an approximate V-shaped form as the theoretical inference, the following process is iteratively performed:

Using the valley point (the point with the minimum standardized residual) on the curve as the boundary, divide the initial set of candidate homologous pixels into two subsets, and take the subset including the real homologous pixels as a new initial set of candidate pixels (supposing that it has *n* groups of pixels). ② If *n* ≠ *m*, compute standardized residuals and draw distribution curve for the new set of *n* groups of candidate pixels again. ③ Judge whether the distribution curve of the new set of candidate pixels still obeys the approximate V-shaped form, if yes, perform step ① again; if not, or the total number of pixels in the new set do not change, stop the iteration.

According to the characteristic of V-shaped curve, on the standardized residual distribution curve obtained in each iteration above, the point with the minimum standardized residual should be located near the middle position of all pixels. Furthermore, since two points cannot form a V-shaped curve hence will stop the iteration, the total number *n* of candidate homologous pixels at the last iteration in each searching image should be two (one of which is the real homologous pixel). This value of *n* is much smaller than that of initial *m*. As the total number of candidate homologous pixels decreases gradually in the iterative process, it is considered as the iterative refinement of the searching range of homologous pixels based on the positional distribution pattern among candidate pixels. In this iterative refinement process, for each searching image, the output results of each iteration before the last are the row and column numbers of two endpoints of the searching range and the position of the real homologous pixel on either the left or right side of the pixel with the minimum standardized residual, and the output of the last iteration is two candidate pixels.

Experiment **II**: According to the iterative refinement of the searching range of homologous pixels in experiment **I**, the total number *n* of candidate homologous pixels corresponding to the matching pixel in the *k*th searching image *I*_*k*_ is two. So the average value of the row and column numbers (*r*_*i*_*, c*_*i*_) of these *n* candidate pixels is used as the row and column numbers (*r*^*k*^, *c*^*k*^) of the final homologous pixel in the image *I*_*k*_: ${r}^{k}=\frac{{\sum }_{i=1}^{n}{r}_{i}}{n}; {c}^{k}=\frac{{\sum }_{i=1}^{n}{c}_{i}}{n}$, and this process of determining the homologous pixel is considered as a new object-side multi-view matching based on the positional distribution pattern among candidate homologous pixels. The reason for adopting Average operation rather than Max or Min operation is as follows: either the Max operation or the Min operation includes the real homologous pixel, the accuracy of this operation for different matching pixel is not guaranteed, and its image-space precision of the homologous pixel is 1 pixel or 0. Whereas, the Average operation can ensure that the image-space precision is 0.5 pixel.

Therefore, for the 100 matching pixels in experimental images, the new object-side multi-view matching was firstly carried out to obtain the row and column numbers (*r*^*k*^_*j,*O_, *c*^*k*^_*j,*O_) (*k* = 1, 2; *j* = 1, 2,…, 100) of homologous pixels in each searching image. Then based on the image-space matching results, object-space 3D coordinates (*X*_*j,*O_, *Y*_*j,*O_, *Z*_*j,*O_) were figured out according to the principle of multi-view forward intersection. Secondly, utilizing obtained image-space/object-space matching results and corresponding ground truth, image-space row error *∆r*^*k*^_*j*_ and column error *∆c*^*k*^_*j*_ and object-space plane error ∆*P*_*j*_ and elevation error ∆*E*_*j*_ of matching pixels were computed by equations: ${\Delta r}_{j}^{k}={r}_{j,\mathrm{O}}^{k}-{r}_{j}^{k}; {\Delta c}_{j}^{k}={c}_{j,\mathrm{O}}^{k}-{c}_{j}^{k}$, ${\Delta P}_{j}=\sqrt{{\left({X}_{j,\mathrm{O}}-{X}_{j}\right)}^{2}+{\left({Y}_{j,\mathrm{O}}-{Y}_{j}\right)}^{2}}; {\Delta E}_{j}={Z}_{j,\mathrm{O}}-{Z}_{j}$. The curves of image-space and object-space matching errors of all matching pixels were respectively drawn. Thirdly, to verify that the object-side multi-view matching method does not depend on image-side grayscale information, original searching images were randomly adjusted in grayscale, brightness and hue with Photoshop (mainly used to simulate condition such as strong/weak light, repetitive/weak texture, etc.), and were used again in object-side multi-view matching. Image-space and object-space errors (*∆r*^*k*^_*j*_, *∆c*^*k*^_*j*_) and (*∆P*_*j*_, *∆E*_*j*_) of matching results were also recalculated. Lastly, to compare the object-side matching method with traditional image-side matching method, image-side matching based on NCC was carried out for the adjusted searching images to obtain matching results (*r*^*k*^_*j,*I_,*c*^*k*^_*j,*I_) and (*X*_*j,*I_, *Y*_*j,*I_, *Z*_*j,*I_), and corresponding image-space and object-space errors (*∆r'*^*k*^_*j*_, *∆c'*^*k*^_*j*_) and (*∆P'*_*j*_, *∆E'*_*j*_) were also computed.

### Experimental results

The proposed method of analyzing the positional distribution pattern among candidate homologous pixels in multi-view images was realized using the Visual C#.NET 2015 program language, and tested with real aerial images according to the above experimental design. The computer configurations for algorithm verification are: ThinkPad T580 laptop, Win10 64-bit operating system, Intel CORE i7 CPU with 1.99 GHz, and 8G RAM. In the following results, all displayed images were generated by our own program, and all statistical graphs were generated by Microsoft Excel 2016.

#### Results of experiment I

**Figure 7:**
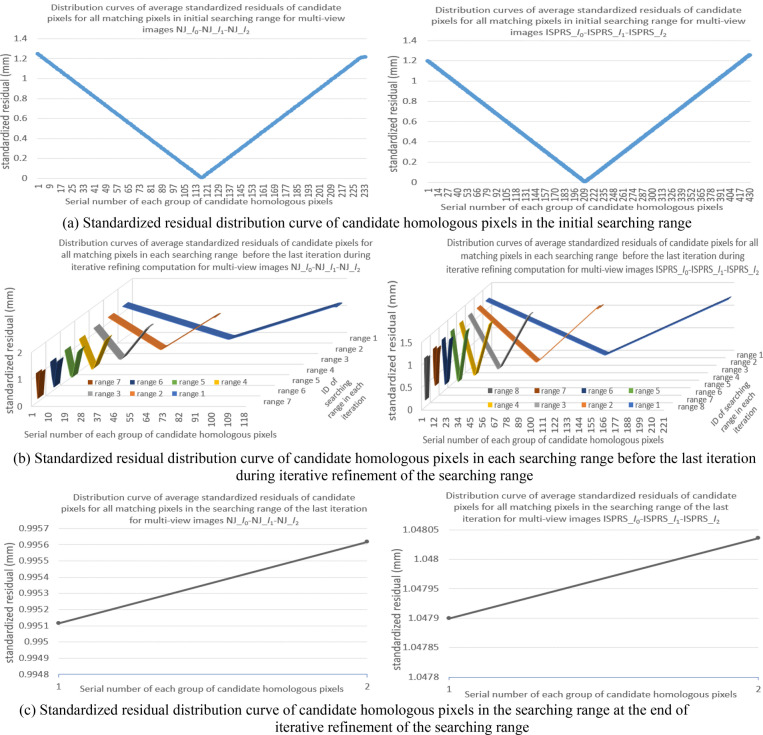
The experimental results of positional distribution form among candidate homologous pixels for all matching pixels in Nanjing and ISPRS multi-view images (left figures and right figures are the results for images NJ_*I*_0_-NJ_*I*_1_-NJ_*I*_2_ and images ISPRS___*I*_0_-ISPRS___*I*_1_- ISPRS___*I*_2_ respectively, and all these figures were created by Microsoft Excel 2016).

Using the mode of multi-view images *I*_0_-*I*_1_-*I*_2_, the experiment **I** were carried out for the two types of experimental images. For the 100 matching pixels in Nanjing and ISPRS experimental images respectively, corresponding standardized residual distribution curves of initial *m* groups of candidate homologous pixels in the initial searching range are shown in Fig. 7a. After processed by the iterative refinement of the searching range, standardized residual distribution curves before the last iteration are shown in Fig. 7b, and the residual distribution curves at the last iteration are shown in Fig. 7c.

In Fig. 7, the horizontal axis represents the serial number of each group of candidate pixels (the maximal total number of candidate pixels for each of the 100 matching pixels was used as the boundary), the vertical axis represents the standardized residual of each group of candidate pixels, and the third axis perpendicular to the horizontal axis in the plane represents the serial number of each searching range during the iterative refinement of the searching range (the searching range at the *i*th iteration was labeled as “range *i*”).

Need to be noted, to effectively display the standardized residual distribution form of each groups of candidate pixels in each searching range for all matching pixels during the iterative refinement process, the standardized residual corresponding to each group serial number in Fig. 7 is the average of standardized residuals of candidate pixels with this group serial number. These average standardized residuals can better express the whole positional distribution form among candidate homologous pixels during the iterative refinement process.

**Figure 8:**
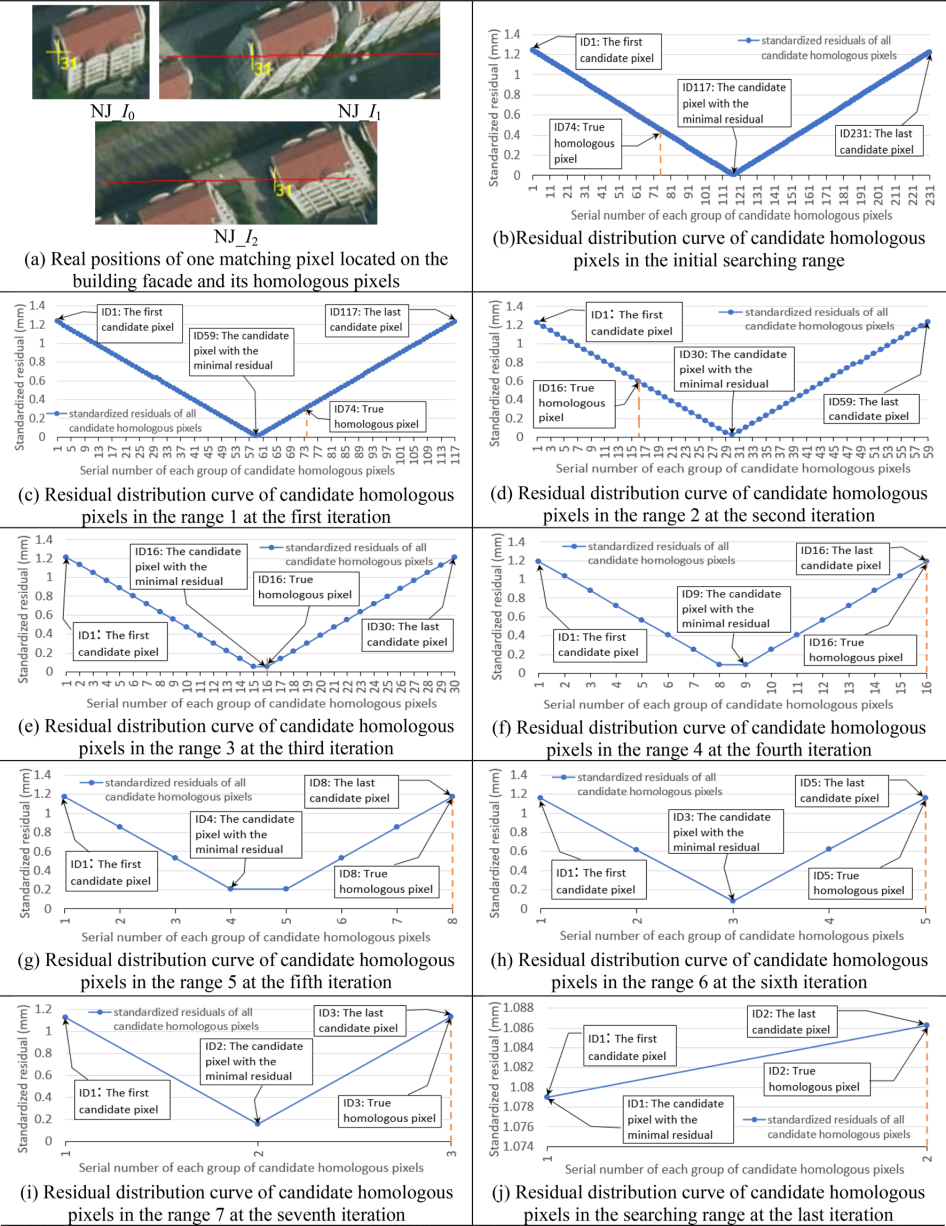
The experimental results of positional distribution form among candidate homologous pixels for one matching pixel located on the building facade in Nanjing experimental images NJ_*I*_0_-NJ_*I*_1_-NJ_*I*_2_ (Graphs in (b) to (j) were created by Microsoft Excel 2016).

**Figure 9:**
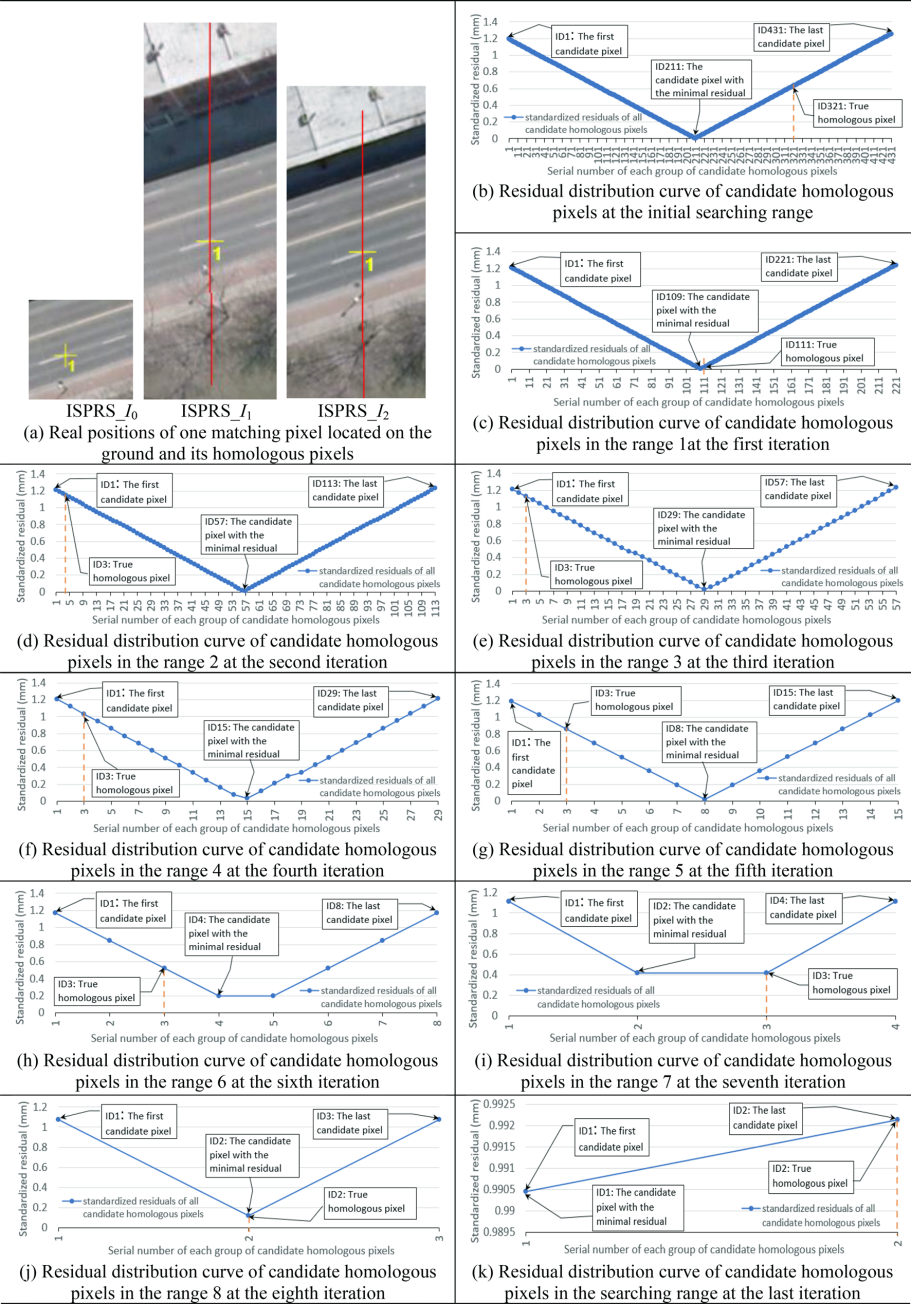
The experimental results of positional distribution form among candidate homologous pixels for one matching pixel located on the ground in ISPRS experimental images ISPRS_*I*_0_-ISPRS_*I*_1_-ISPRS_*I*_2_ (Graphs in (b) to (k) were created by Microsoft Excel 2016).

Furthermore, to clearly demonstrate the positional distribution form among candidate homologous pixels for singular pixel, two matching pixels located on building facade and ground from the two experimental data were taken as examples, and corresponding experimental results were displayed in detail in Figs. 8 and 9. In these figures, the red line in each searching image is the homologous epipolar line defined by the approximate object-space elevation range (that is the initial searching range); and in each sub-figure of the standardized residual distribution curve, each blue dot represents a group of candidate pixels; serial numbers of the first group of candidate pixels, last group of candidate pixels, the group of candidate pixels with the minimum residual and the group of real homologous pixels are all marked with note boxes; and the vertical red dotted line indicates the position of the group of real homologous pixels.

#### Results of the experiment II

**Figure 10:**
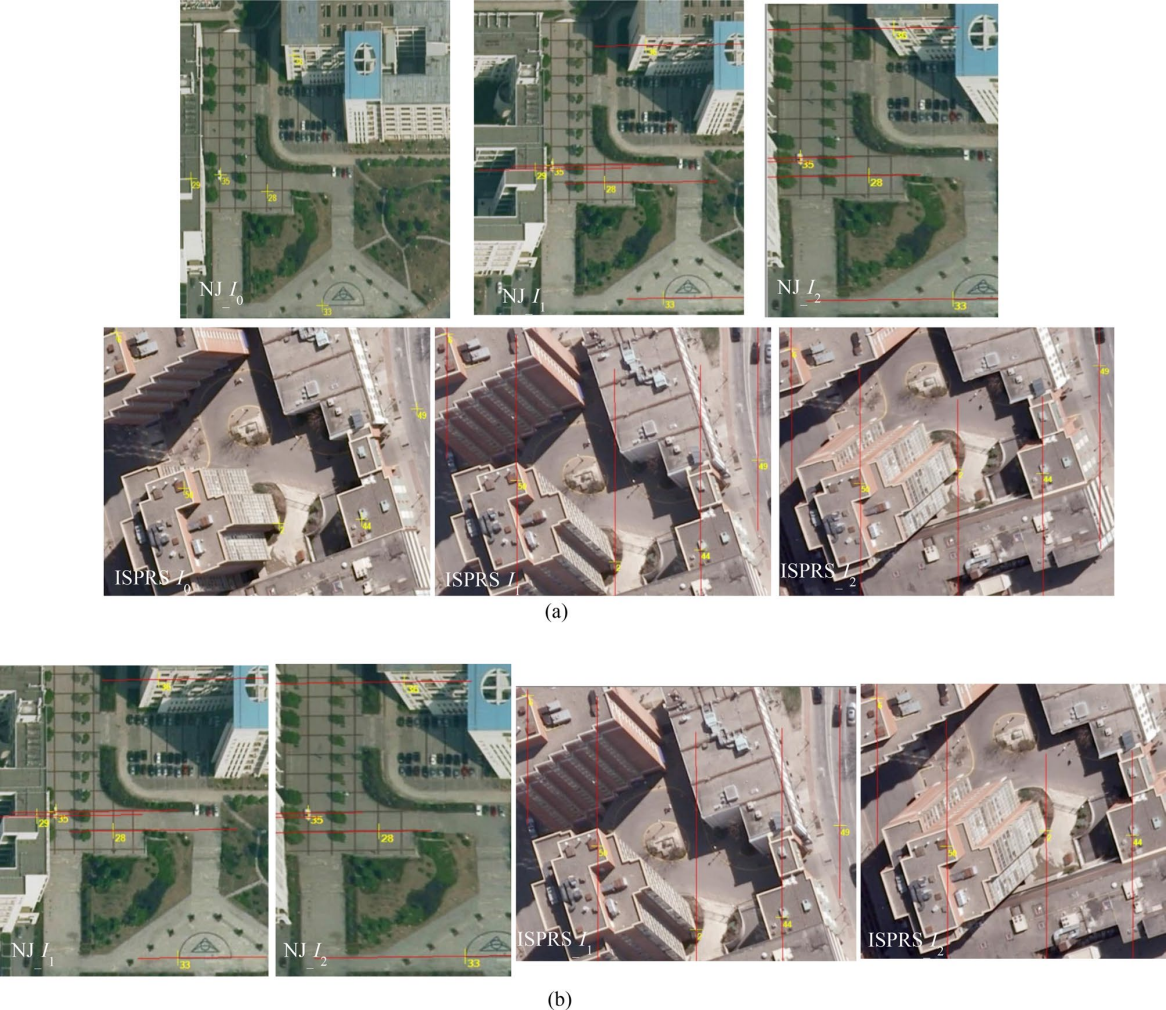
The image-space experimental results of object-side multi-view matching method based on positional distribution pattern among candidate homologous pixels for original Nanjing and ISPRS images: NJ_*I*_0_-NJ_*I*_1_-NJ_*I*_2_ and ISPRS_*I*_0_-ISPRS_*I*_1_-ISPRS_*I*_2_ (All displayed images in this figure were generated by our own program realized using the Visual C#,NET 2015). (**a**) The real positions of partial matching pixels in the base image *I*_0_ and their homologous pixels in two searching images *I*_1_ and *I*_2_ for the two types of experimental data (local area). (**b**) Homologous pixels obtained by object-side multi-view image matching in two searching images for the two types of experimental data (local area)

According to the design of the experiment **II**, part of the 100 matching pixels and corresponding image-space experimental results of the new object-side multi-view matching method are shown in Fig. 10(sub-figure (a) shows positions of partial matching pixels in the base image and corresponding real homologous pixels in the two searching images, and sub-figure (b) shows image-space positions of homologous pixels obtained by the object-side multi-view matching method.).

**Figure 11:**
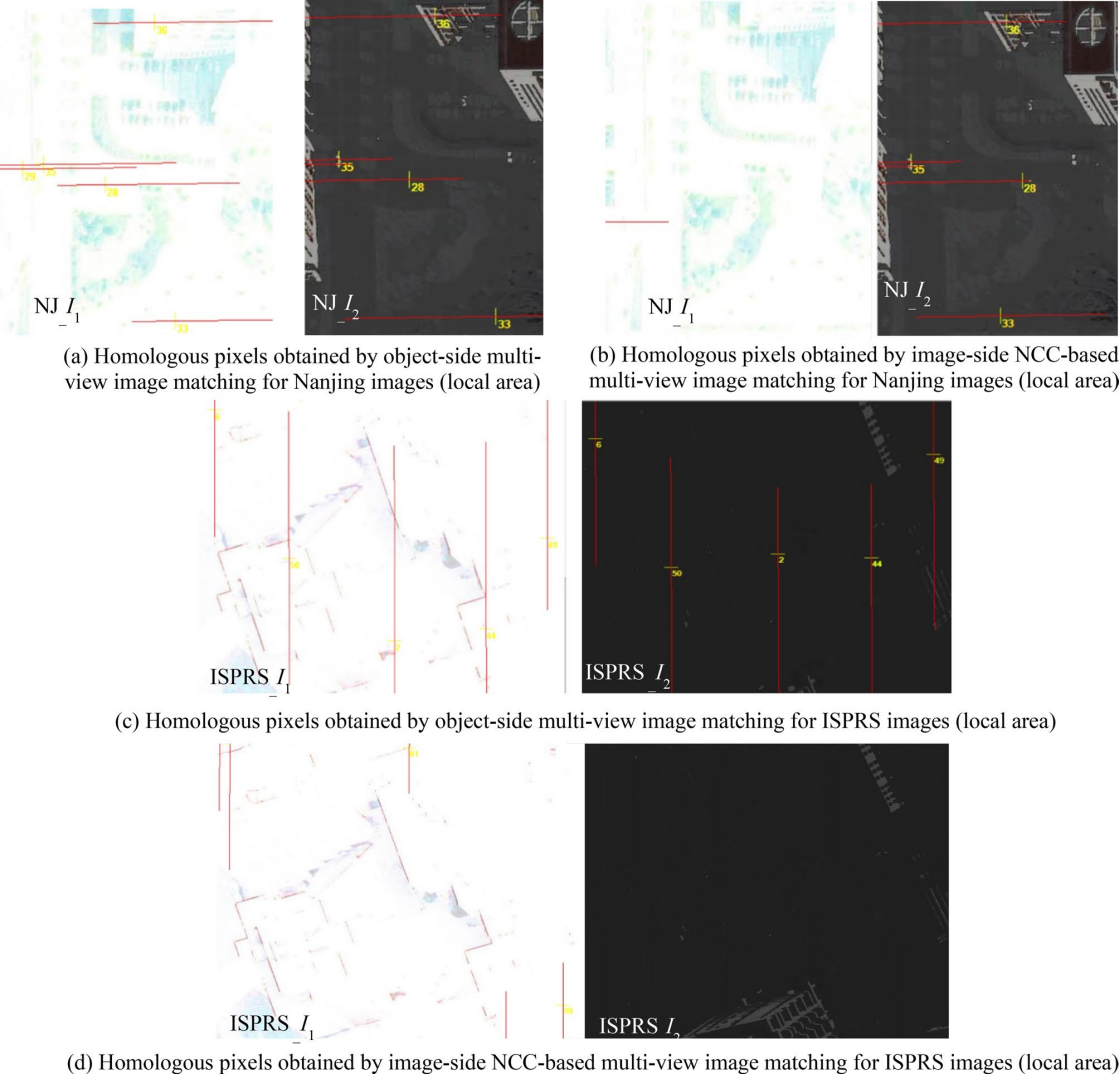
The comparative image-space experimental results of object-side and image-side multi-view matching methods for grayscale adjusted Nanjing and ISPRS images: NJ_*I*_0_-NJ_*I*_1_-NJ_*I*_2_ and ISPRS_*I*_0_-ISPRS_*I*_1_-ISPRS_*I*_2_ (All displayed images in this figure were generated by our own program realized using the Visual C#,NET 2015).

After the grayscale information of Nanjing and ISPRS searching images are adjusted, image-space experimental results of the object-side multi-view matching method for the same matching pixels are respectively shown in Fig. 11a,c. As a contrast, corresponding experimental results of an image-side NCC-based matching method are respectively shown in Fig. 11b,d.

**Figure 12:**
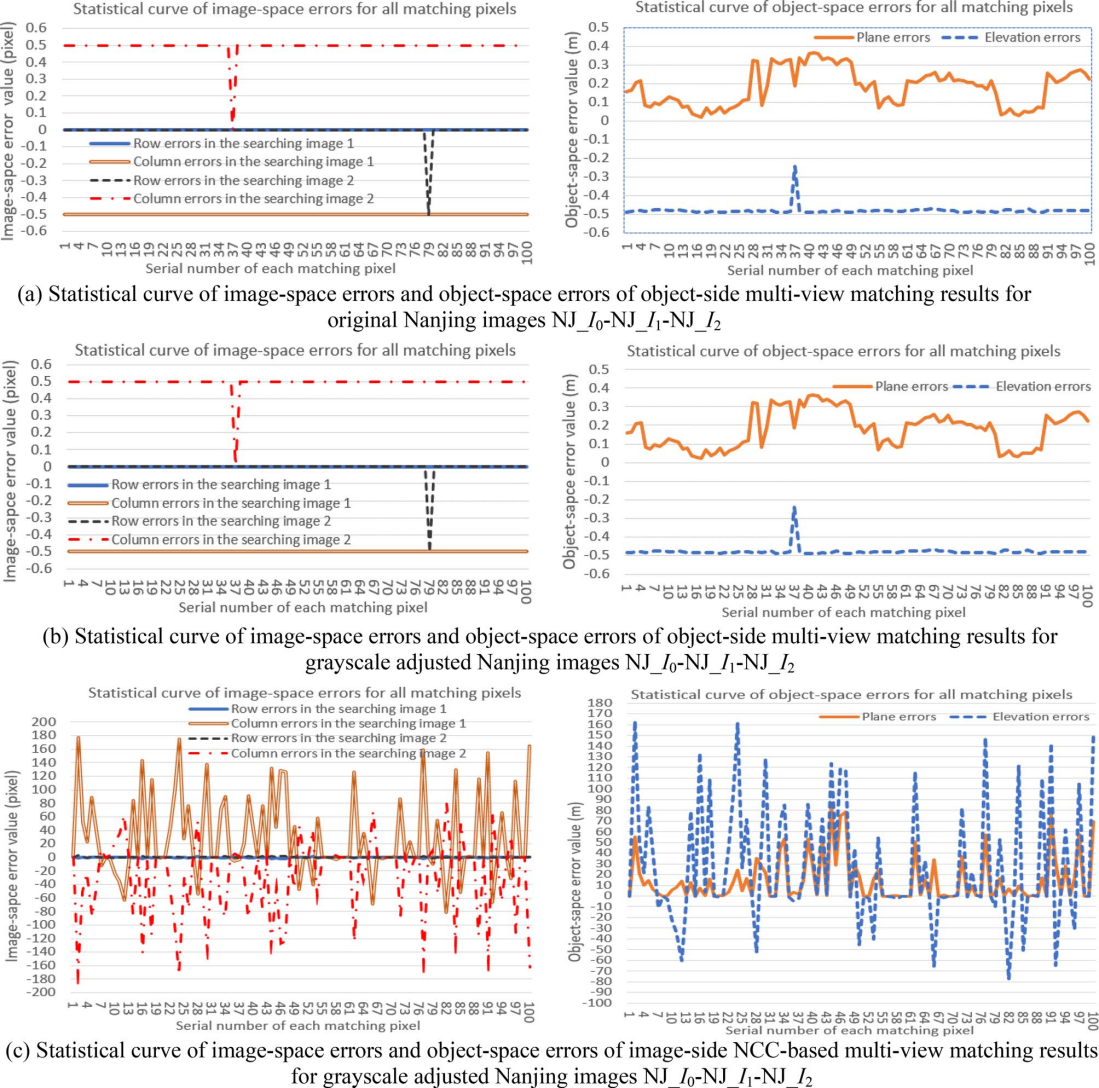
The comparative results of statistical curve of image-space errors (left column) and object-space errors (right column) of different multi-view matching results for original and grayscale adjusted Nanjing images (All these graphs were created by Microsoft Excel 2016).

**Figure 13:**
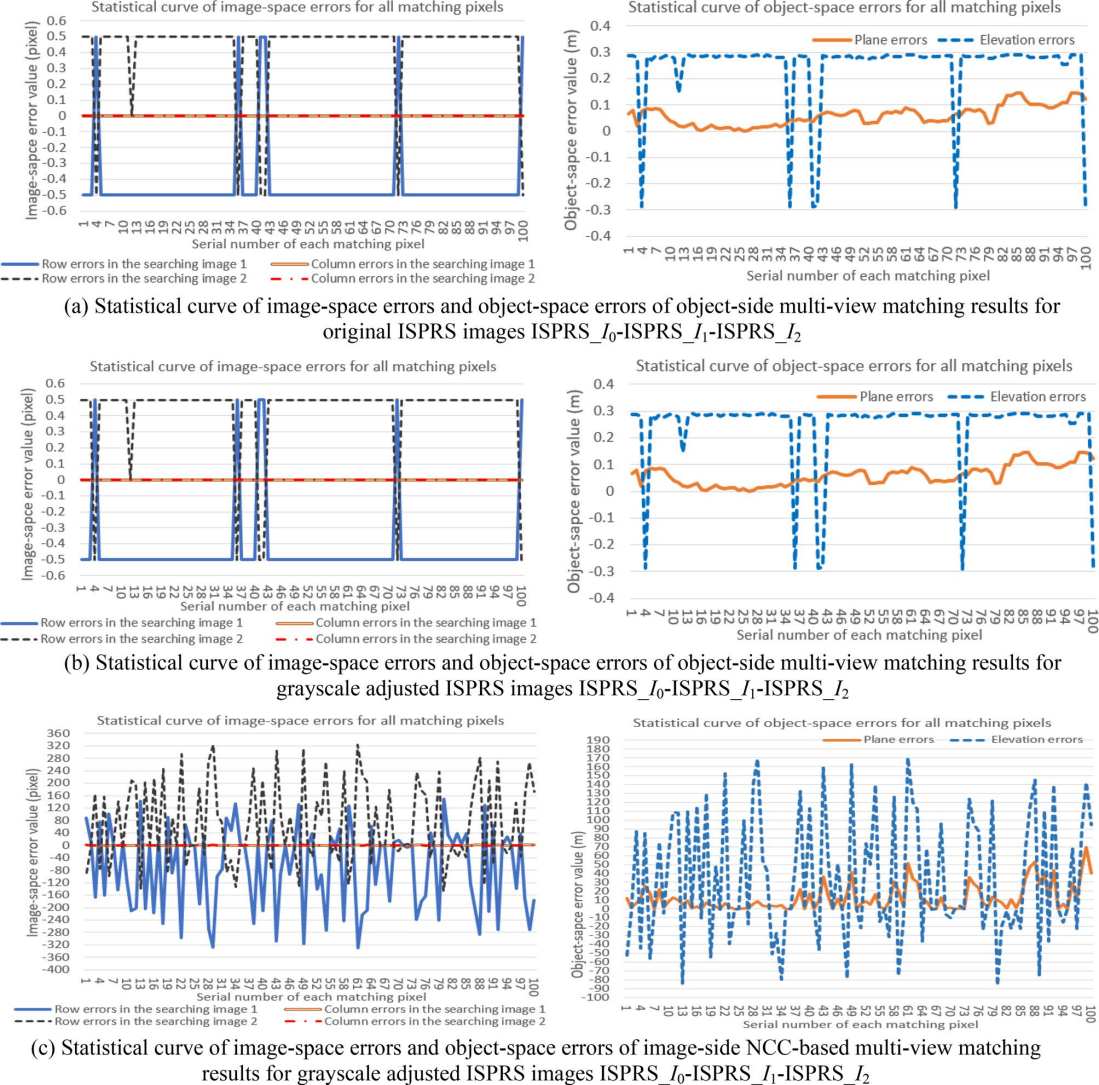
The comparative results of statistical curve of image-space errors (left column) and object-space errors (right column) of different multi-view matching results for original and grayscale adjusted ISPRS images (All these graphs were created by Microsoft Excel 2016).

In addition, to clearly display comparative experimental results of the object-side matching method and traditional image-side matching method for original and grayscale adjusted images of the two types of experimental data, corresponding statistical curves of errors (*∆r*^*k*^_*j*_, *∆c*^*k*^_*j*_) and (*∆P*_*j*_, *∆E*_*j*_) of the object-side matching results, and (*∆r'*^*k*^_*j*_, *∆c'*^*k*^_*j*_) and (*∆P'*_*j*_, *∆E'*_*j*_) of the image-side NCC-based matching results, are respectively shown in Figs. 12 and 13. In these figures, sub-figures (a) and (b) respectively show statistical curve of image-space errors (*∆r*^*k*^_*j*_, *∆c*^*k*^_*j*_) and object-space errors (*∆P*_*j*_, *∆E*_*j*_) of object-side multi-view matching results for original and grayscale adjusted experimental images, and sub-figure (c) shows corresponding statistical curve of image-space errors (*∆r'*^*k*^_*j*_, *∆c'*^*k*^_*j*_) and object-space errors (*∆P'*_*j*_, *∆E'*_*j*_) of the image-side NCC-based matching results for grayscale adjusted experimental images.

Furthermore, to intuitively display the quantitative effect of the application of positional distribution pattern among pixels in multi-view image matching, the rule that the image-space row and column errors of one matching result are both less than or equal to 1 pixel (∆*r* ≤ 1 &∆*c* ≤ 1) is used as the judgment criterion of matching accuracy. The quantitative comparison results of matching accuracy, image-space and object-space errors for object-side and image-side multi-view matching methods are listed in the Table 3. In addition, the running time of the object-side matching method for the two types of experimental data is respectively 125.126 s and 909 s. As the total number of candidate pixels in the initial searching range and the number of iterative refinement process are not identical, the running time for the two types of experimental data are different. Although the efficiency is not high, it is not the emphasis of this study, which is whether the positional distribution pattern is valid for confirming the exact homologous pixels in multi-view matching and robust to the distortion of image-side grayscale information. In future work, we will try to adopt acceleration techniques such as parallel computing, GPU acceleration and code optimization in the algorithm implementation process to improve running efficiency of our method.

**Table 3:** Quantitative comparison results of object-side and image-side multi-view matching methods in image matching for two types of experimental images.

Image type		The total number of matching pixels	Matching method	Matching accuracy (%)	Image-space error of matching results (pixel)		Object-space error of matching results (m)	
					The range of ∆*r*	The range of ∆*c*	The range of ∆*P*	The range of ∆*E*
Original images	NJ_*I*_0_-NJ_*I*_1_-NJ_*I*_2_	100	Image-side method	Used as the ground truth				
			Object-side method	100	[− 0.5, 0]	[− 0.5, 0.5]	(0.02, 0.4)	(− 0.5, − 0.2)
	ISPRS_*I*_0_- ISPRS_*I*_1_- ISPRS_*I*_2_		Image-side method	Used as the ground truth				
			Object-side method	100	[− 0.5, 0.5]	[0, 0]	(0, 0.15)	(− 0.3, 0.3)
Searching images adjusted in grayscale	NJ_*I*_0_-NJ_*I*_1_-NJ_*I*_2_	100	Image-side method	36	[− 2, 3]	[− 174, 177]	[0, 86)	(− 78, 163.3)
			Object-side method	100	[− 0.5, 0]	[− 0.5, 0.5]	(0.02, 0.4)	(− 0.5, − 0.2)
	ISPRS_*I*_0_- ISPRS_*I*_1_- ISPRS_*I*_2_		Image-side method	18	[− 330, 325]	[− 1, 2]	[0, 70)	(− 86.4, 170.3)
			Object-side method	100	[− 0.5, 0.5]	[0, 0]	(0, 0.15)	(− 0.3, 0.3)

### Analysis and discussion

Seen from above experimental results in Figs. 7, 8, 9 of experiment I, for any matching pixel in the base image of Nanjing multi-view images or ISPRS benchmark images (no matter where it is located), its candidate homologous pixels in the searching range on each searching image obey the following positional distribution pattern: First, when the total number of candidate homologous pixels is more than two, the standardized residual distribution curve of all candidate pixels presents an approximate V-shaped form. Second, on the V-shaped distribution curve, the valley point with the minimum residual basically locates at the middle of all candidate pixels, and the real homologous pixel lies on either the left or right of the valley point (including this valley point).

Utilizing the V-shaped distribution pattern among candidate homologous pixels, the iterative refinement of searching range in the searching image can be realized. After refinement, the total number of candidate homologous pixels in each searching image will be reduced from initial hundreds (depending on the size of the initial searching range) to final two adjacent pixels containing the real homologous pixel. However, for different images or different matching pixels in the same image, the iterative times and the total number of candidate pixels in each iteration may be different.

Furthermore, positional distribution pattern among candidate homologous pixels, which is verified in experiment I, can be used to develop a new object-side multi-view image matching method. This new method does not need to construct 2D image-side matching windows and use the grayscale or feature information of pixels in the windows to compute the matching similarity at all, but only needs the standardized residual of each candidate homologous pixel obtained by the adjustment of pseudo object-space coordinates of the matching pixel. Seen from above experimental results in Figs. 10, 11, 12, 13 and Table 3 of experiment II, as the object-side matching method does not depend on the grayscale information of the image, its matching results are identical to original results when matching images have nonlinear variation in the grayscale (even the eyes can no longer distinguish objects in the images). As a contrast, the matching accuracy of the traditional image-side matching method will decrease greatly in this condition. The reasons for choosing image-side NCC-based matching method for comparison are as follows. Firstly, the NCC-based method can obtain better matching result than SAD or SSD-based method for images with grayscale distortion. Secondly, the experimental matching pixels and their homologous pixels are obtained by the NCC-based method interactively. If the new object-side matching method is more robust than NCC-based method under the circumstance of gray-scale distortion, it can strongly prove the validity of the proposed method.

In addition, the quantitative experimental results in Table 3 show that the new object-side matching method can obtain a matching error of less than or equal to 0.5 pixel in the image space, a plane error of less than 0.4 m and an elevation error of less than 0.5 m in the object space. These object-space errors have met the plane accuracy requirement for large scale digital terrain mapping in China, as well as the elevation accuracy requirement of regions with a terrain slope greater than 2°, according to the Chinese surveying and mapping industry standard of “Digital products of fundamental geographic information 1:500, 1:1000 and 1:2000 digital line graphs” (numbered as CH/T 9008.1-2010) (In this industry standard, for 1:500 digital line graphs, depending on the types of terrain, the limitations of the plane error are 0.6 m (terrain slope < 6°) and 0.8 m (terrain slope ≥ 6°), and the limitations of the elevation error are 0.4 m (terrain slope < 2°), 0.8 m (2° ≤ terrain slope < 6°) , 1.0 m (6° ≤ terrain slope < 25°) and 1.4 m (terrain slope ≥ 25°)).

## Conclusion

Considering whether there is a specific positional distribution pattern among candidate homologous pixels of the matching pixel in multi-view images, this paper proposes a new conception of pseudo object-space coordinates and its adjustment computation model, and a new method of studying the positional distribution pattern based on the surveying adjustment theory. Actual aerial images were used to carry out experiments and verify the proposed method. Through experiments, it has been found: for candidate homologous pixels in the searching range corresponding to the matching pixel in multi-view images, using the standardized residual of each candidate pixel obtained by the adjustment of pseudo object-space coordinates as the indicator, their image-space positions appear a special V-shaped distribution pattern. Furthermore, this positional distribution pattern can be used to realize the iterative refinement of the searching range of the homologous pixel, which can reduce candidate homologous pixels from initial hundreds to final two.

In addition, based on the special positional distribution pattern among candidate homologous pixels, this paper designs a full object-side multi-view image matching process. In this process, the construction of 2D image-space matching windows and the computation of matching similarity between matching windows with image-side grayscale information are all not needed. It only uses the positional distribution pattern among candidate pixels to iteratively confirm the homologous pixels in multi-view images corresponding to the matching pixel. Whereas, the image-side grayscale information is a required input for traditional region-based matching methods. Thus, the proposed object-side matching method has an obvious distinction from traditional matching methods. The experimental results verify that the object-side matching method can achieve an image-space matching accuracy of 0.5 pixel, and an object-space positioning accuracy that meets the precision requirement of large-scale digital mapping in China.

To our knowledge, this is the first report of positional distribution pattern among candidate homologous pixels in multi-view images in the field of photogrammetry, and the proposed research method on this pattern is expected to provide a new thought for resolving the problems that existing image-side multi-view image matching methods face, i.e., low reliability of matching results in difficult matching regions (such as large geometric distortion, obvious radiometric difference, weak texture or repetitive texture, etc.) at a mechanism level. Thus, the research of this paper has great significance on enriching the existing theories and methods of multi-view image matching, and has potential application value in improving the computational efficiency and accuracy of dense matching for large-scale multi-view images.

However, the study object of this paper is still limited to central projective aerial images, and further study is needed in the following two aspects in the future. First, the proposed method should be applied to more types of multi-view images, for example, push-broom multiline space images and close-range images. Second, morphological characteristics (such as slope, length, etc.) of the standardized residual distribution curve of candidate pixels should be further studied to design a quantitative index for accurately indicating whether the real homologous pixel is located to the left side or right side of the valley point on the distribution curve. If this quantitative index can be found, a new object-side multi-view image matching method based on the positional distribution pattern of pixels, and does not depend on the image quality, can be really realized.
